# Serum Hepcidin Concentration in Individuals with Sickle Cell Anemia: Basis for the Dietary Recommendation of Iron

**DOI:** 10.3390/nu10040498

**Published:** 2018-04-17

**Authors:** Juliana Omena, Cláudia dos Santos Cople-Rodrigues, Jessyca Dias do Amaral Cardoso, Andrea Ribeiro Soares, Marcos Kneip Fleury, Flávia dos Santos Barbosa Brito, Josely Correa Koury, Marta Citelli

**Affiliations:** 1 Instituto de Nutrição, Universidade do Estado do Rio de Janeiro, Rio de Janeiro 20550-900, Brazil; omenaju@gmail.com (J.O.); claudiacople@gmail.com (C.d.S.C.-R.); jessycadiaxs@hotmail.com (J.D.d.A.C.); barbosaflavia@bol.com.br (F.d.S.B.B.); jckoury@gmail.com (J.C.K.); 2 Faculdade de Ciências Médicas, Universidade do Estado do Rio de Janeiro, Rio de Janeiro 20550-900, Brazil; andrearsoares@hotmail.com; 3 Faculdade de Farmácia, Universidade Federal do Rio de Janeiro, Rio de Janeiro 21941-590, Brazil; marcos.fleury@yahoo.com.br

**Keywords:** sickle cell anemia, hepcidin, iron overload

## Abstract

Dietary iron requirements in patients with sickle cell disease (SCD) remain unclear. SCD is a neglected hemoglobinopathy characterized by intense erythropoietic activity and anemia. Hepcidin is the hormone mainly responsible for iron homeostasis and intestinal absorption. Intense erythropoietic activity and anemia may reduce hepcidin transcription. By contrast, iron overload and inflammation may induce it. Studies on SCD have not evaluated the role of hepcidin in the presence and absence of iron overload. We aimed to compare serum hepcidin concentrations among individuals with sickle cell anemia, with or without iron overload, and those without the disease. Markers of iron metabolism and erythropoietic activity such as hepcidin, ferritin, and growth differentiation factor 15 were evaluated. Three groups participated in the study: the control group, comprised of individuals without SCD (C); those with the disease but without iron overload (SCDw); and those with the disease and iron overload (SCDio). Results showed that hepcidin concentration was higher in the SCDio > C > SCDw group. These data suggest that the dietary iron intake of the SCDio group should not be reduced as higher hepcidin concentrations may reduce the intestinal absorption of iron.

## 1. Introduction

Sickle cell disease (SCD) is an inherited hemoglobinopathy caused by the substitution of glutamic acid by valine at the 6th position of the beta globin chain. This modification induces the formation of hemoglobin S (Hb S), causing red blood cells to acquire the sickle shape and, consequently, leading to chronic hemolysis and the occurrence of vessel occlusion phenomena, pain episodes, and injury of organs and tissues [1].

Blood transfusions are administered in order to treat manifestations of the disease, improve the capacity of oxygen transport and minimize hemolysis, as in splenic sequestration crises [2], or to prevent complications and disease progression, as in the prevention of stroke in children [3,4]. Despite the benefits, regular transfusions can lead to iron overload, since each unit of transfused blood contains about 200–250 mg of this mineral [5,6]. In healthy people, the body’s iron content is around 4 g [7], while chronically transfused individuals can store 5 g to 10 g per year [8]. With the progression of iron overload, iron becomes potentially toxic, due to its tendency to catalyze the formation of reactive oxygen species, consequently leading to oxidative stress and culminating in cellular damage [9].

Hepcidin—a polypeptide hormone formed by a sequence of 25 amino acids, from the transcription of the HAMP (hepcidin antimicrobial peptide) gene—plays a significant role in iron homeostasis through its binding to ferroportin, a protein responsible for the export of iron from various cell types, especially enterocytes and macrophages of the reticuloendothelial system [10]. The hepcidin–ferroportin complex is internalized by these cells, and ferroportin undergoes degradation, blocking iron output and consequently leading to a reduction in the absorption of intestinal iron and its bioavailability [11,12].

The intense but ineffective erythropoiesis and anemia inherent to the SCD are factors that potentially lead to reduced hepcidin concentration [13], leading to increased intestinal absorption of this micronutrient. In contrast, the characteristic inflammatory feature as well as the increased serum iron concentration can induce the transcription of this hormone [14], reducing iron absorption.

As the factors that activate and inhibit hepcidin synthesis may be simultaneously present in patients with SCD, the common nutritional approach used by health professionals to treat individuals with iron overload is the restriction of the most abundant food sources of this mineral, such as meats, mainly viscera, and legumes. However, these actions may also reduce the bioavailability of other minerals, such as zinc, present in foods that are also sources of iron. Patients with the disease usually have reduced plasma zinc concentrations [15]. 

Hepcidin has not yet been sufficiently studied in SCD. A few existing studies present inconclusive data regarding hepcidin concentration, as most studies do not differentiate patients with SCD in relation to the presence or absence of iron overload, nor do they compare these concentrations with healthy control groups [16,17,18,19,20,21,22,23]. Thus, it is difficult to define the appropriate iron-related nutritional care for this group as data regarding the behavior of hepcidin are limited. Hence, this study aimed to compare the serum hepcidin concentration in people with sickle cell anemia, with or without iron overload, and to a control group without the disease. Markers of iron metabolism and erythropoietic activity were also evaluated.

## 2. Materials and Methods

### 2.1. Study Participants

Adult patients aged 18–59 years old, of both genders, with sickle cell anemia (Hb SS genotype) were recruited from the hematology and hemolytic anemia outpatient clinics, respectively, at the Pedro Ernesto University Hospital and Arthur Siqueira de Cavalcanti State Institute of Hematology (Hemorio) (Rio de Janeiro, Brazil). The control group was composed of healthy volunteers without sickle cell anemia (Hb AA genotype).

Participants with sickle cell trait, those with other hemoglobinopathies and hematological diseases, pregnant women, patients with SCD who had been hospitalized, and/or those who received blood transfusions 15 days prior to blood collection were excluded from this study.

In patients without SCD (control group), those with serum ferritin <10 ng/mL (for women) and <20 ng/mL (for men) were excluded from the study. Participants who used medications for treatment of diabetes and hypo/hyperthyroidism were also excluded.

Participants were divided into three groups: sickle cell disease with iron overload (SCDio) group, consisting of patients with SCD and serum ferritin ≥1000 ng/mL; sickle cell disease without iron overload (SCDw) group, those with SCD and ferritin <1000 ng/mL; and the control (C) group, composed of individuals without SCD. To compare the results without differentiating the presence of iron overload, the SCDio and SCDw groups were merged and named sickle cell disease group (SCD). Serum ferritin ≥1000 ng/mL was adopted based on the cut-off point referenced by the study by Porter and Garbowski [24]. 

All study participants received and signed the informed consent form before taking part in the study. The study was approved by the Research Ethics Committees of Pedro Ernesto University Hospital (number: 758.174) and Arthur de Siqueira Cavalcanti State Institute of Hematology (number: 391/15).

### 2.2. Anthropometry and Nutritional Assessment

Body mass and height were determined using a 0.1 kg portable precision scale (Filizola, São Paulo, Brazil). The participants’ body mass index (BMI) was calculated (body mass/height^2^). Participants with BMI between 18.5 kg/m^2^ and 24.9 kg/m^2^ were considered eutrophic [25].

### 2.3. Blood Sampling and Analysis

Participants’ blood was collected by venipuncture in the morning for routine hematological and biochemical analyses. For hematological analysis, the blood was collected in tubes with anticoagulant ethylenediamine tetraacetic acid (EDTA). For serological and biochemical analyses, collection tubes with clot activator gel were used to extract the serum.

Except for the samples collected in EDTA tubes, the others were centrifuged at 700× *g* for 10 min, aliquoted into microtubes, and stored in a freezer at a temperature of −80 °C until analysis. 

### 2.4. Hematological Measurements and Biochemical Analysis

Hemoglobin, hematocrit, and leukocytes were analyzed using the automated counter Horiba^®^ Pentra 60 C^+^ (Horiba ABX Diagnostics, Pentra 60 C^+^, Montpellier, France), which combines the principles of electrical impedance, flow cytometry, cytochemistry, and spectrophotometry.

To confirm the genotypes of the study participants, the determination and quantification of normal hemoglobin and variants were performed through ion-exchange high-performance liquid chromatography using the Variant^TM^ equipment (Bio-Rad Laboratories, Hercules, CA, USA).

Serum ferritin levels were analyzed using a commercially available enzyme-linked immunosorbent assay (ELISA) kit (Symbiosys, ALKA Tecnologia^®^, São Paulo, Brazil).

The serum concentration of bioactive hepcidin-25 (DRG Instruments GmbH, Marburg, Germany), growth differentiation factor 15 (GDF-15) (Sigma Aldrich Inc., Saint Louis, MO, USA) and Interleukin 6 (IL-6) (Merck Millipore, Darmstadt, Germany) were also analyzed using ELISA. 

Serum iron and total iron-binding capacity (TIBC) were analyzed by a colorimetric method using Ferrozine*^®^*(Labtest, Belo Horizonte, Brazil). Transferrin saturation (TS) index is calculated as follows: serum iron/TIBC× 100.

The lactate dehydrogenase (LDH) analysis was performed by continuous ultraviolet kinetics method (pyruvate-lactate method) using the Labmax Plenno automatic analyzer (Labtest, Belo Horizonte, Brazil).

Analyses were performed according to the manufacturers’ instructions. 

### 2.5. Statistical Analysis

The distribution of variables was analyzed for normality using the Shapiro–Wilk test. As most of the variables did not present a Gaussian distribution, we chose to use non-parametric tests. 

Measures of central tendency and dispersion were expressed as median and interquartile ranges (1st–3rd quartile). In the descriptive analysis, data were expressed as frequencies (*n*) and percentages (%).

The Mann–Whitney U test was used to assess the differences of continuous variables between the two groups and the Kruskal–Wallis test for the three study groups (*p* < 0.05). Subsequently, the Mann–Whitney U test with Bonferroni correction (*p* < 0.017) was used to compare the pairs of groups.

The statistical analysis was performed using the Statistical Package for Social Science software (IBM SPSS^®^ Inc., version 22.0, Chicago, IL, USA).

## 3. Results

A total of 158 individuals participated in the study; 115 met the inclusion criteria, 72 had SCD (54.2%, male), and 43 were included as a control group (41.9%, male).

The median age of patients with SCD was 29.5 years (Interquartile Range, IQR: 18–59 years), while the control group was 26.0 years (IQR: 19–54 years), denoting homogeneity among individuals. However, the median age of the SCDio group was higher than the other groups (38.5; IQR: 22–59 years).

Most of the participants with SCD (62.5%) were classified as eutrophic, whereas those in the control group had similar percentages of eutrophy and overweight (48.8%) (Table 1).

Biochemical data obtained from a single group of individuals with SCD, without separation of the groups into SCDw and SCDio, are presented in Table 2. As expected, individuals with SCD presented lower hematocrit and hemoglobin concentrations when compared with the control group. However, the SCD group had significantly lower serum hepcidin concentrations. Regarding other markers of iron metabolism, ferritin, serum iron, and TS levels were higher in the SCD group than in the control group, while the TIBC values were higher in the control group than in the SCD group. Regarding parameters related to hemolysis and erythropoietic activity, the SCD group had higher median values for both LDH and GDF-15 than the control group. This result was expected, since SCD is a disease that presents with intense hemolysis and erythropoiesis.

Regarding serum ferritin levels, participants with SCD were then divided into SCDio group (individuals with SCD and ferritin ≥1000 ng/mL; *n* = 14) and SCDw group (individuals with SCD and ferritin <1000 ng/mL; *n* = 58) (Table 3). The concentrations of hemoglobin and hematocrit markers in the SCDw and SCDio groups were lower than in the control group (*p* < 0.001), in agreement with what had already been observed in the SCD group. These parameters did not differ between the SCDw and SCDio groups. The same was observed for leukocytes, which presented higher medians in both SCDw and SCDio groups (10.7 × 10^3^/mm^3^ vs. 10.9 × 10^3^/mm, respectively) than the control group (5.8 × 10^3^/mm^3^) but did not present statistical difference when compared among them.

Furthermore, the three groups had significantly different serum hepcidin concentrations. The SCDio group presented the highest levels of this hormone (11.6 ng/mL) in comparison to the other groups, while the SCDw group had the lowest median values (4.2 ng/mL), both in relation to SCDio and in the control group (7.2 ng/mL). This result could only be evidenced by the separation of the groups into SCDio and SCDw (Table 3). 

The concentration of serum iron in the SCDio group was higher than that in the control group, due to the excessive iron load to which they were exposed due to blood transfusions. However, no difference was observed between the SCDw and SCDio groups and neither between the SCDw group and control group.

The values of TIBC and TS variables (Table 3) in the control group were lower than those presented by the SCDio and SCDw groups, the same as observed in Table 2.

LDH levels were higher in the SCDw and SCDio groups than in the control group (Table 3). However, the GDF-15 marker presented a statistically significant difference between the three groups analyzed, with higher concentrations in the SCDio group (1643.7 pg/mL) than in the SCDw (1227.3 pg/mL) and control groups (504.8 pg/mL).

SCD is characterized by the presence of low-grade chronic inflammation, which may be evidenced by interleukin-6 (IL-6) and leukocyte concentrations (Table 2). For this reason, we evaluated whether the differences in ferritin concentrations between the control group and the SCD groups would be maintained when corrected for the leukocyte (ferritin/leukocyte ratio) value. The comparative analysis showed that the ferritin/leukocyte ratio is higher in the SCD groups (Table 2 and Table 3).

## 4. Discussion

Transfusional iron overload has frequently been reported in studies of sickle cell anemia. Although the data on its prevalence are still limited, it is known that the increase in the life expectancy of individuals with sickle cell anemia may lead to a greater number of blood transfusions and, consequently, an increase in cases of iron overload.

This study showed that patients with SCD and iron overload had serum hepcidin concentrations higher than those in patients with SCD without iron overload and individuals of the control group. However, when all patients with SCD were merged in a single group, serum hepcidin concentration in the SCD appeared to be lower than that in the control group.

Previous studies reported various hepcidin concentrations in patients with sickle cell disease, which can be explained by the heterogeneity of the studied groups. Most of the studies analyzed the sickle cell group without considering the signs of iron overload observed among the participants; additionally, other studies compared the hepcidin concentrations of people with sickle cell disease with that of other hematological diseases [16,17]. To our knowledge, this is the first study to evaluate hepcidin concentration in the two groups of adults with SCD, with the main difference being the presence or absence of iron overload, identified by serum ferritin values.

The findings of this study corroborate those found by Nemeth [14], who showed increased urinary hepcidin excretion in two patients with SCD and signs of iron overload when compared with healthy individuals.

In some previous studies that considered the SCD group as a whole, hepcidin concentrations in these patients were lower [18], equal [19,20,21,22], or even higher [23] than in the control group. Such variations are probably due to the fact that several of the studies do not distinguish the participants regarding their transfusion behavior, that is, they include individuals who never received blood transfusion and the polytransfused ones, and/or do not distinguish iron overload by ferritin concentration (>1000 ng/mL).

In this study, the history and frequency of blood transfusions of the participants varied; all patients had already been transfused, and about 36.1% of them reported having had an average of 10 or more transfusions (simple or blood exchange) (data not shown).

As observed in our study, the analysis of all individuals with SCD who present characteristics so different from each other can interfere in the results found, since the group comprised of patients with SCD presented a serum hepcidin concentration smaller than the one observed in the control group. Thus, the serum hepcidin concentration of the SCDio group was masked and could only be revealed when the separation between groups with and without iron overload was performed. 

These results show that the behavior of hepcidin in SCD may be influenced by various changes in this hemoglobinopathy, whether linked to iron metabolism or factors inherent to the disease itself. Regarding inflammation, as revealed by IL-6 and leukocyte concentrations, it was increased in patients with SCD, with and without iron overload, when compared to individuals in the control group. It is known that inflammation is one of the factors capable of increasing hepcidin concentration. In inflammation, the interaction of IL-6 with its receptor (IL-6R) activates JAK tyrosine kinases, triggering the formation of STAT3 (Signal transducer and transcription activator 3) complexes that bind to the hepcidin promoter in the nucleus [26]. Additionally, obesity seems to be another factor capable of exerting influence on serum hepcidin concentrations [27]. However, in the present study, the percentage of individuals with excess of adiposity was lower in the SCD group. 

Karafin et al. [28] also investigated the relationship between possible factors that could contribute to changes in the hepcidin concentration in SCD and observed that erythropoiesis markers were the strongest factors capable of influencing its serum concentration, followed by serum ferritin. These results were in line with the findings of this study, since the concentration of GDF-15 in the SCD group was 2.5-fold higher than that in the control group, which could explain the reduced hepcidin concentration in the group with SCD. Increase in the concentration of this marker was already expected. In the iron overload group, high levels of iron, identified by the high concentration of serum ferritin, overlapped the elevation of GDF-15 levels, possibly leading to higher hepcidin concentration.

This study revealed the heterogeneity of the characteristics of the SCDw and SCDio groups. The behavior of hepcidin in SCD needs to be interpreted considering the presence or absence of iron overload, since iron regulates the synthesis of several molecules involved in its own homeostasis. 

In many countries, including Brazil, some public policies have been developed to prevent iron deficiency, such as mandatory fortification of wheat and corn flours [29]. Although these actions can help prevent iron-deficiency anemia and are aimed at the entire Brazilian population, the safety of these actions is still unknown for individuals with diseases caused by iron overload such as hereditary hemochromatosis, thalassemia, and SCD. To date, data on intestinal iron absorption in sickle cell disease are limited, making it impossible to guarantee that these actions do not cause harm to this group.

However, some authors have observed iron deficiency in people with SCD. Kassim et al. [30] observed that 13.3% of the SCD participants presented iron deficiency—most of them had never undergone transfusion previously. In this case, the diagnosis of iron deficiency was established if all the following four criteria were present: low serum iron <45 μg/ dL, low transferrin saturation (TS) <16%, high TIBC ≥450 μg/dL, and low MCV for age.

## 5. Conclusions

Differences in hepcidin concentrations between groups may suggest an increase (in the case of the SCD and SCDw groups) or decrease (SCDio) in the intestinal absorption of iron. These data suggest that individuals with iron overload may not need to reduce intake of foods rich in iron. However, these results are not sufficient to the establishment of a nutritional approach to be adopted. Future studies evaluating the intestinal absorption of iron in patients with sickle cell anemia must be conducted to address this question. Regarding the participants in the group without overload, suppression of hepcidin appears to occur possibly due to the increase of erythropoiesis in response to anemia, which is characteristic of the disease and, consequently, could lead to increased intestinal absorption of iron.

## Figures and Tables

**Table 1:** Frequency of the general characteristics of patients with sickle cell disease (*n* = 72) and the control group (*n* = 43).

General Characteristics	Patients with SCD *n* (%)	Control *n* (%)
Gender, male	39 (54.2%)	18 (41.9%)
Underweight	14 (19.4%)	1 (2.4%)
Eutrophic	45 (62.5%)	21 (48.8%)
Overweight	10 (13.9%)	16 (37.2%)
Obese type I	3 (4.2%)	4 (9.3%)
Obese type II	0 (0%)	0 (0%)
Obese type III	0 (0%)	1 (2.3%)

**Table 2:** Comparison of laboratorial parameters between the control and SCD groups (SCDw + SCDio).

Laboratorial Parameters	Control Group *n* = 43		SCD Groups *n* = 72		*p* Value
	Median	IQR (P25–P75)	Median	IQR (P25–P75)	
Hemoglobin (g/dL)	13.5	12.8–14.7	8.0	7.1–9.3	<0.001
Hematocrit (%)	40.1	38.7–44.1	23.6	21.4–28.1	<0.001
Leukocytes (×10^3^/mm^3^)	5.8	4.9–6.6	10.7	7.6–13.9	<0.001
Lymphocytes (%)	37.1	33.0–42.1	38.4	30.8–46.0	0.676
Hepcidin (ng/mL)	7.2	5.6–11.6	5.3	2.5–10.9	0.014
Ferritin (ng/mL)	29.8	17.1–68.5	228.7	69.4–703.5	<0.001
Ferritin/Leukocytes ratio	6.3	3.1–13.2	27.5	7.2–77.0	<0.001
Serum iron (µg/dL)	105.0	69.0–129.0	119.0	92.0–158.8	0.012
TIBC (µg/dL)	333.0	301.0–383.0	293.0	233.3–347.0	0.001
TS (%)	31.4	20.5–41.8	43.1	30.1–62.5	<0.001
LDH (U/L)	349.0	293.0–391.0	818.0	619.8–1198.0	<0.001
GDF-15 (pg/mL)	504.8	396.0–652.4	1299.2	618.4–1553.1	<0.001
IL-6 (pg/mL)	0.0	0.0–2.5	3.8	2.4–8.9	<0.001

Results are expressed as median and interquartile ranges (IQR); P25–P75: 25th–75th percentile. Differences were tested using Mann–Whitney U test, *p* < 0.05. SCD: sickle cell disease groups, SCDw: sickle cell disease without iron overload, SCDio: sickle cell disease with iron overload, TIBC: total iron-binding capacity, TS: transferrin saturation, LDH: lactate dehydrogenase, GDF-15: growth differentiation factor 15, IL-6: interleukin-6.

**Table 3:** Comparison of laboratorial parameters between the control, SCDw, and SCDio groups.

Laboratorial Parameters	Control		SCDw		SCDio		*p* Value
	*n* = 43		*n* = 58		*n* = 14		
	Median	IQR (P25–P75)	Median	IQR (P25–P75)	Median	IQR (P25–P75)	
Hemoglobin (g/dL)	13.5 ^a^	12.8–14.7	8.2 ^b^	7.2–9.4	7.3 ^b^	6.0–8.3	<0.001
Hematocrit (%)	40.1 ^a^	38.7–44.1	24.4 ^b^	21.7–28.3	22.2 ^b^	18.5–26.1	<0.001
Leukocytes (×10^3^/mm^3^)	5.8 ^a^	4.9–6.6	10.7 ^b^	7.2–13.7	10.9 ^b^	9.5–14.6	<0.001
Lymphocytes (%)	37.1 ^a^	33.0–42.1	39.5 ^a^	31.0–47.7	35.0 ^a^	26.7–41.5	0.167
Hepcidin (ng/mL)	7.2 ^a^	5.6–11.6	4.2 ^b^	2.2–7.8	11.6 ^c^	9.8–23.0	<0.001
Ferritin (ng/mL)	29.8 ^a^	17.1–68.5	167.8 ^b^	60.3–436.3	1986.4 ^c^	1379.7–2261.6	<0.001
Ferritin/Leukocytes ratio	6.3 ^a^	3.1–13.2	14.1 ^b^	6.4–33.8	172.7 ^c^	127.0–249.3	<0.001
Serum iron (µg/dL)	105.0 ^a^	69.0–129.0	111.5 ^a,b^	92.0–146.8	158.5 ^b^	97.3–204.0	0.006
TIBC (µg/dL)	333.0 ^a^	301.0–383.0	293.0 ^b^	244.8–349.0	270.5 ^b^	202.8–321.0	0.001
TS (%)	31.4 ^a^	20.5–41.8	38.4 ^b^	29.3–58.0	55.6 ^b^	38.7–81.4	<0.001
LDH (U/L)	349.0 ^a^	293.0–391.0	894.0 ^b^	596.5–1328.5	730.5 ^b^	684.3–997.5	<0.001
GDF-15 (pg/mL)	504.8 ^a^	396.0–652.4	1227.3 ^b^	593.7–1496.7	1643.7 ^c^	1299.8–1702.3	<0.001
IL-6 (pg/mL)	0.0 ^a^	0.0–2.5	3.7 ^b^	2.4–8.3	4.3 ^b^	2.9–15.2	<0.001

Results are expressed as median and interquartile ranges (IQR); P25–P75: 25th*–*75th percentile. Differences between three groups were tested using Kruskal–Wallis test (*p* < 0.05). Post hoc analysis was performed using the Mann–Whitney U test for two groups with Bonferroni correction (*p* < 0.017), different letters indicate statistical difference between groups; SCDw: sickle cell disease without iron overload, SCDio: sickle cell disease with iron overload, TIBC: total iron binding capacity, TS: transferrin saturation, LDH: lactate dehydrogenase, GDF-15: growth differentiation factor 15.
